# Notes on some toad bugs from China (Hemiptera, Heteroptera, Gelastocoridae)

**DOI:** 10.3897/zookeys.759.21627

**Published:** 2018-05-22

**Authors:** Tong-Yin Xie, Guo-Qing Liu

**Affiliations:** 1 College of Agriculture,; 2 Northeast Agricultural University, Harbin 150030, China; 3 Key Laboratory of Soybean Biology, in Chinese Ministry of Education, Northeast Agricultural University, Harbin 150030, China; 4 Institute of Entomology, Nankai University, Tianjin, 300071, China

**Keywords:** Hemiptera, Gelastocoridae, *Nerthra*, China

## Abstract

The three species of *Nerthra* Say, 1832 (Hemiptera: Heteroptera: Gelastocoridae) occurring in China are reviewed. Dorsal habitus photographs of the two species, *Nerthra asiatica* (Horváth, 1892) and *Nerthra indica* (Atkinson, 1889), are provided, accompanied by illustrations of male genitalic structures and female ventral aspect of posterior abdominal segments. The male of *Nerthra asiatica* is recorded and reviewed for the first time.

## Introduction

Toad bugs (Gelastocoridae) are a remarkable group of aquatic bugs (Nepomorpha) which are derived from aquatic ancestors and have become secondarily terrestrial (Hebsgaard et al. 2004). Gelastocoridae contains three recent genera and approximately 103 species distributed worldwide, but much more prevalent in the tropics (Polhemus 1995). It is divided into two subfamilies, Gelastocorinae and Nerthrinae. Recent Gelastocorinae (two genera) are reported in only in America, from southern Canada to north Argentina (Štys and Jansson 1988; Chen et al. 2005), but there is one fossil species, *Gelastocoris curiosus* Poinar & Brown, 2016 described from Burmese amber (Poinar and Brown 2016). The Nerthrinae includes one fossil genus, *Cratonerthra* Martins-Neto, 2005 with two species (Ruf et al. 2005), and one recent genus, *Nerthra* Say, 1832, currently including 92 valid recent species, of which nine species occur in south-eastern Asia west of Wallace line, and three species present in China (Kment and Jindra 2008, Xie and Liu 2013, Faúndez and Ashworth 2015).

## Material and methods

**Figure 1.**
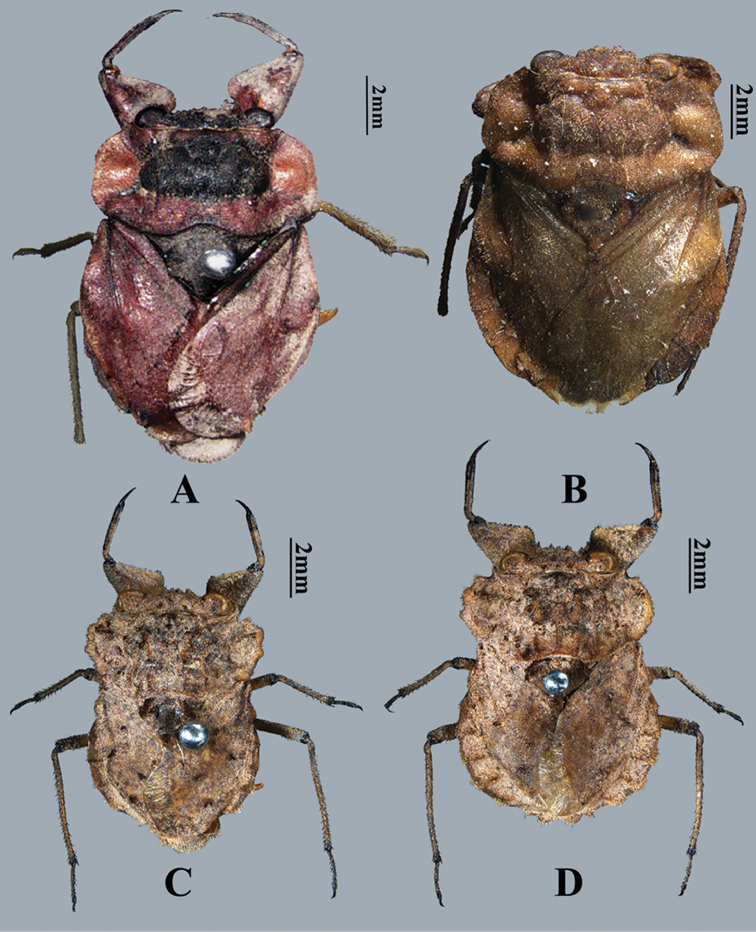
Dorsal habitus of *Nerthra* spp. **A** *N. asiatica* (Horváth) (♂) **B** *N. asiatica* (♀) **C** *N. indica* (Atkinson) (♂) **D** *N. indica* (♀).

The male genitalia were examined in glycerol and illustrated using a Zeiss Discovery V8 microscope. All measurements are given in millimetres (see Table 1). The digital photographs of specimens (Fig. 1A–D) were taken with a Zeiss Discovery V20 camera. All the studied specimens are deposited in the Institute of Entomology, Nankai University (NKUM), Tianjin, China.

**Table 1.** Measurements of *Nerthra* species.

**Species and sex**	**Range**	**Body length**	**Body width**	**Head length**	**Head width**	**Pronotum length**	**Pronotum width**
*Nerthra asiatica*							
Male (*N* = 1)		12.3	8.2	0.8	4.8	3.3	7.2
Female (*N* = 4)	min	11.6	8.1	0.9	4.6	0.9	7.7
	max	12.3	8.9	1.2	4.8	1.3	8.2
	average	11.8	8.5	1.1	4.7	1.2	8.0
*Nerthra indica*							
Male (*N* = 31)	min	8.7	5.9	0.4	3.9	2.3	6.1
	max	9.2	6.3	0.6	4.2	2.7	6.5
	average	9.0	6.1	0.5	4.0	2.6	6.3
Female (*N* = 48)	min	9.6	6.6	0.4	3.9	2.4	6.7
	max	10.3	7.8	1.1	4.5	3.1	7.8
	average	9.9	7.3	0.7	4.3	2.9	7.5
Nerthra macrothorax*							
Male	min	7.9	6.0	–	–	–	5.9
	max	–	–	–	–	–	–
	average	–	–	–	–	–	–
Female	min	9.2	6.7	–	–	–	6.8
	max	10.6	8.2	–	–	–	8.2
	average	9.9	7.45	–	–	–	7.5

* these measurements are from Polhemus and Polhemus 2012 and Todd 1955.

## Systematics

### Nerthra asiatica

Taxon classificationAnimaliaHemipteraGelastocoridae

(Horváth, 1892)

Figs 1A, B ; 2A–G

Mononyx asiaticus Horváth, 1892: 136.
Mononyx grossus Montandon, 1899: 395 (syn. Kiritshenko 1926: 226); Distant 1906: 16; Oshanin 1909: 956; Oshanin 1912: 89; Kiritshenko 1926: 226; Wu 1935: 559.
Nerthra asiatica : Todd 1955: 349; Todd 1957: 154; Nieser and Chen 1992: 5; Polhemus 1995: 24; Thirumalai 1998: 192; Bal and Basu 2003: 542; Kment and Jindra 2008: 191; Xie and Liu 2013: 6.

#### Material examined.

**CHINA: Sichuan Province**: 1♂, Mount Emei [峨眉山], 29.58N, 103.41E, 24. IV. 1962, Bai-juan CHEN leg.; 1♀, Ya’an [雅安], 29.98N, 103.01E, 4. VII. 1963, alt. 600–900m, Jiang XIONG leg.; **Hubei Province**: 1♀, Wufeng Tujia Autonomous County [五峰土家族自治县], 30.20N, 110.67E, 10. VII. 1999, alt. 1000m, Chuan-ren LI leg.; 1♂, National Natural Reserve of Xingdou Mountain [星斗山国家级自然保护区], 30.14N, 109.00E, 30. VII. 1999, alt. 840–900m, Chuan-ren LI leg.; **Xizang (Tibet) Autonomous Region**: 1♀, Mêdog county [墨脱县], 29.33N, 95.34E, alt. 800m, VIII. 1984, Tan HE leg.

#### Redescription.

Body large size for the genus. Body dorsally brown with scutellum slightly darker than rest (Fig. 1A–B). Ventral surface dark brown, the bases of the middle and hind legs with a few patches of yellowish brown.

*Head*. Apical tubercle absent, lateral and superapical tubercles small, irregular in shape, not sharply pointed.

*Thorax*. Pronotum widest at transverse furrow, a little narrower than abdomen; lateral margins of pronotum parallel or nearly so, anterior and posterior margin weakly sinuate; surface coarsely granulate. Scutellum elevated, apex slightly lobed, with tumescences at the middle of the lateral margins. Hemelytra not extending to the end of the abdomen, membrane well developed; embolium with the basal half of the lateral margin nearly straight, not expanded laterally at middle. Connexivum greatly expanded laterally in females. Bristles short or moderately long, clavate, slightly curved, bristles in rows and clumps on hemelytra and in clumps on scutellum and pronotum.

**Figure 2.**
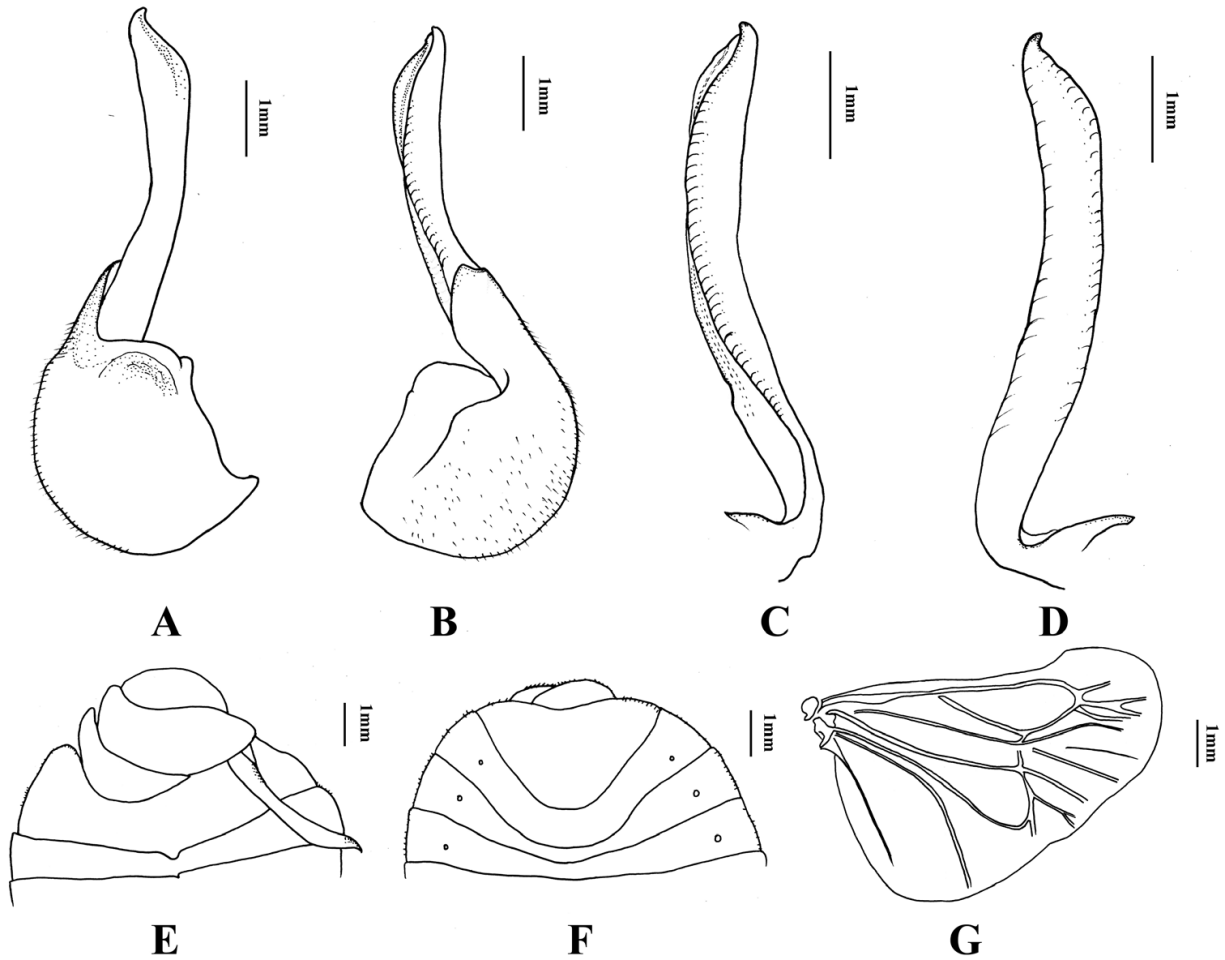
*Nerthra asiatica* (Horváth). **A–B** Genital capsule in different views **C–D** Right paramere in different views **E** Ventral view of posterior abdominal segments of male **F** Female subgenital plate **G** hind wing.

*Abdomen*. Abdominal V-IV sternites of male mostly asymmetrical, ninth sternite rather oval, wider than long, not as long as eighth sternite; seventh sternite sternite about half as long as eighth sternite; fifth sternite very short medially (Fig. 2E). In female, abdomen nearly symmetrical. Lobes of ovipositor slightly projecting posteriorly; posterior margin of last visible abdominal sternite triangularly emarginate (Fig. 2F).

#### Distribution.

China (Sichuan Province, Hubei Province, Xizang (Tibet) Autonomous Region), India (Todd 1955, Kment and Jindra 2008).

#### Remarks.

This is the first time the male has been described. In the shape of the pronotum it would seem to be closely related to *N. spissa* (Distant, 1911), but the right paramere (Fig. 2C–D) of these two species is different. *Nerthra spissa* has a rather large male clasper for the size of the insect, nearly straight, cylindrical, abruptly narrowed to point at apex, twisted, and the aedeagal furrow obliquely crossing the basal half of clasper. This species differs from *N. indica* (Atkinson, 1889) by the its larger body size and the shapes of the tubercles of the head, the lateral margin of the pronotum, the hind wing (Fig. 2G), and the structures of male and female genitalia.

The holotype is a female from China: Flumen Poi-ho (G. N. Potanin)’ [= Sichuan, Gar Qu (= Pai Ho River)] (Kiritshenko 1926; Todd 1955; Polhemus 1995). The paratype of *Mononyx grossus* Montandon in the Francis Huntington Snow Entomological Collection at the University of Kansas was labelled ‘Thibet (Mou-Pin)’ [= Sichuan, Ya’an (= Mou-ping country)] (Todd 1955). This species found in Mêdog county is reported from Xizang (Tibet) Autonomous Region for the first time.

### Nerthra indica

Taxon classificationAnimaliaHemipteraGelastocoridae

(Atkinson, 1889)

Figs 1C, D ; 3A–G

Mononyx indicus Atkinson, 1889: 345; Montandon 1899: 394; Distant 1906: 15; Maxwell-Lefroy 1909: 709; Paiva 1919: 372.
Mononyx projectus Distant, 1911: 310 (syn. Todd 1955: 405).
Mononyx turgidulus Distant, 1911: 311 (syn. Todd 1961: 94).
Nerthra turgidula : Todd 1955: 406; Bal and Basu 2003: 542.
Nerthra indica : Todd 1955: 405; Todd 1961: 93; Nieser 1977: 298; Todd 1977: 216; Lansbury 1988: 189; Nieser and Chen 1992: 5; Bal and Basu 2003: 542; Kment and Jindra 2008:, 195; Xie and Liu 2013: 6.
Nerthra arunachalensis Thirumalai, 1998: 190; syn. Kment and Jindra 2008: 195.

#### Material examined.

**CHINA: Jiangxi Province**: 1♂, Jinggang Mountain [井冈山], 26.75N, 114.29E, 27. VII. 2002, Wan-liang ZHANG & Jian-hua DING leg.; **Fujian Province: 1**♀, ChongAn [崇安], 27.75N, 118.03E, VI. 1982, Qiang HE leg.; 1♂, Jianning Country [建宁县](26.83N, 116.84E), 26. IX. 2002; Wan-liang ZHANG leg.; 1♂, 2♀, Natural reserve of Jiangshi [将石自然保护区], 27.12N, 117.26E, 13. VIII. 2011, Zhen YE leg.; **Guangxi Zhuang Autonomous Region**: 1♂, Longsheng country [龙胜县], 25.80N, 110.01E, 14. VI. 1963, Si-kong LIU leg.; 1♀, Yao Autonomous County of Jinxiu[金秀瑶族自治县], 24.13N, 110.19E, 23. IX. 1981, Collector unknown; 1♀, Shengtang Mountain of Jinxiu[金秀圣堂山], 24.96N, 110.12E, alt. 900m, 18. V. 1999, Fu-sheng HUANG leg.; 1♂, 1♀, Defu of Napo country[那坡德孚], 23.39N, 105.83E, alt. 1350m, 21. VI. 2000, Jian YAO leg.; 1♀, Beidou of Napo country[那坡北斗], 23.04N, 105.93E, alt. 550m, 22. VI. 2000, Jian YAO leg.; 1♀, Jingxi Diding Autonomous Region[靖西底定自治区], 23.09N, 105.99E, alt. 1000–1700m, 23. VI. 2000, Jian YAO leg.; 1♂, Tiantang mountain of Rong country[玉林市容县黎村天堂山], 22.58N, 110.73E, alt. 730–740m, 17. VIII. 2009, Bo CAI & Ke-long JIAO leg.; **Guizhou Province**: 1♂, 7♀, Maolan National Nature Reserve[茂兰国家级自然保护区], 23.43N, 103.02E, 30. VII. 2013, Tong-yin XIE & Fu-xia HE leg.; **Yunnan Province**: 1♀, Pingbian Miao Autonomous county[屏边苗族自治县], 22.98N, 103.68E, alt. 1500m, 28. V. 1996, Wen-jun BU leg.; 1♂, 1♀, Mengkuan river of Mengla country[勐腊县勐仑镇勐宽河], 21.45N, 101.56E, 18.VIII.2010, Jing WANG leg.; 15♂, 24♀, Menglun town of Mengla country[勐腊县勐仑镇], 21.94N, 101.25E, alt. 534m, 4. VIII. 2010, Kai DANG leg.; 1♀, Nangun river of Cangyuan country[沧源县班洪乡南滚河保护区], 23.29N, 99.10E, alt. 534m, 6. V. 2011; Zhen YE leg.; **Xizang (Tibet) Autonomous Region**: 1♂, Mêdog county[墨脱县], 29.33N, 95.34E, alt. 1100m, VIII. 1984, Tan HE leg. 2♂, Mêdog county suburb[墨脱城郊], 29.30N, 95.36E, alt. 1100m, 15. VIII. 2003, Huai-jun XUE & Xin-pu WANG leg.; 1♂, 2♀, Beibeng town of Mêdog county[墨脱背崩县城], 29.24N, 95.18E, alt. 780–1100m, 13. VIII. 2003, Huai-jun XUE & Xin-pu WANG leg.; 1♀, Mêdog county-108K[墨脱县城-108K], 29.33N, 95.33E, alt. 880–1100m, 16. VIII. 2003, Huai-jun XUE & Xin-pu WANG leg..

#### Redescription.

Body middle sized for the genus. Body dorsally brown, with variable yellowish or other marking, often obscured by muddy crust. Scutellum slightly darker than rest of dorsal surface (Fig. 1C–D). Body sculpture, outlines of the pronotum, hemelytra and abdomen very variable.

*Head*. Apex of head with four tubercles, one at the apex is not visible in the dorsal view, the others sometimes rather indistinct (Fig. 1C–D).

*Thorax*. The lateral margins of the pronotum markedly asymmetrical, pronotum about as wide at anterior third as at the level of the transverse furrow. Scutellum elevated, tumescent laterally and at apex, with curved ridge paralleling sinuosity of posterior margin of pronotum. The outline of the ovipositor was the same and the ventral submarginal tumescences on the last visible abdominal sternite absent. Hemelytra not quite reaching end of abdomen in the females, membrane well developed; embolium narrow at base, dilated before middle, anterior portion and apex of dilation more or less rounded. Ventral surface and the apex of the fore, middle, and hind legs dark brown.

*Abdomen*. Abdomen greatly expanded laterally in females. Bristles mostly short and clavate, groups of long black bristles on basal tumescences and median part of pronotum. Abdominal sternites of male mostly asymmetrical, but nearly symmetrical in female. Lobes of ovipositor asymmetrical slightly lobed and projecting posteriorly. Ninth sternite wider than long, not as long as eighth sternite; seventh sternite sternite about half as long as eighth sternite; fifth sternite very short medially. Right paramere swollen apically and stick out at middle.

#### Remarks.

**Figure 3.**
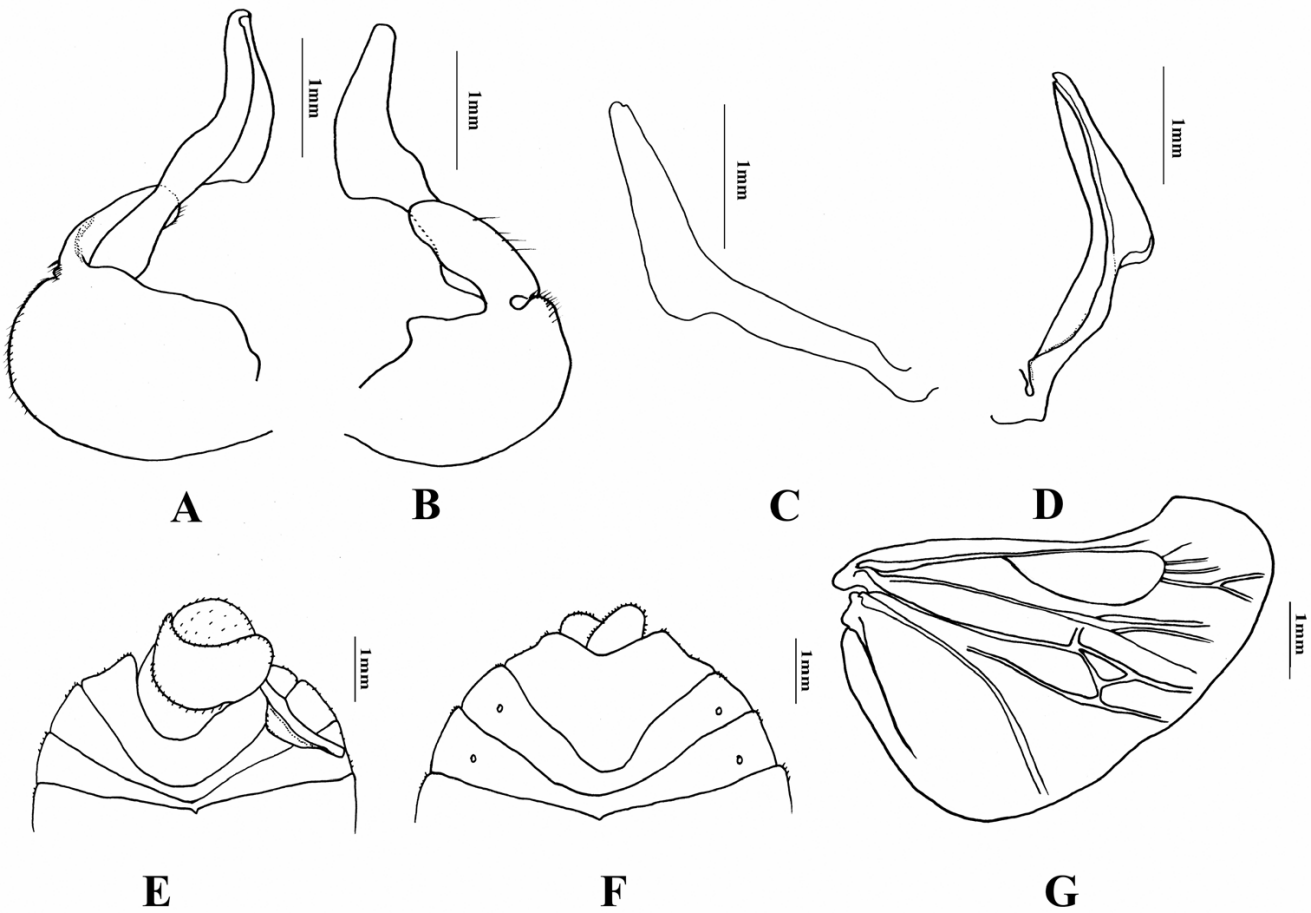
*Nerthra indica* (Atkinson). **A–B** Genital capsule in different views **C–D** Right paramere in different views **E** Ventral view of posterior abdominal segments of male **F** Female subgenital plate **G** hind wing.

Body shape most closely related to *N. lobata* (Montandon, 1899) from which it may be separated by the male genitalia shape (Fig. 3A–D), the smaller ovipositor lobes which are less projecting, and the lack of lateral submarginal tumescences of the last visible abdominal sternite in the females.

#### Distribution.

China (Jiangxi, Fujian, Guangxi Zhuang Autonomous Region, Sichuan, Guizhou, Yunnan, Xizang (Tibet) Autonomous Region), India, Nepal, Vietnam, Laos (Todd 1955; Kment and Jindra 2008).

### Nerthra macrothorax

Taxon classificationAnimaliaHemipteraGelastocoridae

(Montrouzier, 1855)

Galgulus macrothorax Montrouzier, 1855: 110.
Scylaecus macrothorax : Stål 1861: 201.
Peltopterus macrothorax : Stål 1863: 408; Montandon 1899: 779; Kirkaldy 1906: 150; Esaki 1928: 75; Sonan 1934: 21; Hoffmann 1941: 44; Miyamoto 1953: 35, Miyamoto 1954: 28.
Nerthra macrothorax : Todd 1955: 414; Todd 1957: 157; Todd 1959: 63; Todd 1960: 172; Todd 1961, 93; Polhemus 1995: 24; Chen et al. 2005: 47; Nieser and Chen 2005: 308; Kment and Jindra 2008: 203; Polhemus and Polhemus 2012: 357, Xie and Liu 2013: 6; Sano 2016: 31.

#### Description

(from Todd 1955). Body light brown, front of head provided with five large, rounded tubercles, four of which are flatted on top and densely covered with short clavate bristles; ocelli absent. Pronotum greatly expanded laterally; lateral margins converging anteriorly, subparallel for posterior half; posterior angle projecting obliquely posterolateral, rather pointed; posterior margin with five concavities.

Scutellum rather small, apex narrowed, basal portion depressed, inclining to apex which is the most elevated part. Hemelytra entirely coriaceous, fused together, extending slightly beyond end of abdomen, large longitudinal carinae present; base of embolium greatly expanded laterally. Connexivum broadly expanded laterally in both sexes. Entire body covered with short, broadly clavate bristles, bristles pale and especially dense on pronotum and on the elevations of the head.

Abdominal sternites of female nearly symmetrical except for posterior margin of last sternite, which is slightly emarginated, but with apex slightly convex just below the lobes of the ovipositor, the latter somewhat rounded and the left one overlapping the right. Abdominal sternites of male rather small, last visible abdominal sternite wider than long, nearly twice as long as seventh sternite, which has the right side elongate, spatulate.

Clasper of male rather sickle-shaped, but nearly straight, very slightly enlarged at apex then tapering to a blunt point.

#### Notes.

During the daytime this species hides in wet mud or sand, or under stones or plant debris (Chen et al. 2005). Nieser and Chen (2005) observed these toad bugs burrowing in the sand on a beach in the south of Taiwan. In view of its inability to fly, its wide distribution is attributed to dispersion by drift on plant debris (Todd 1960). The authors have not seen this species, and distribution data for this species was collected from the published literature.

#### Distribution.

China (Taiwan), Japan, Philippines, Malaysia, Indonesia, Australia (Kment and Jindra 2008).

## Supplementary Material

XML Treatment for Nerthra asiatica

XML Treatment for Nerthra indica

XML Treatment for Nerthra macrothorax
